# Body temperature as a dynamic host-response biomarker in sepsis: trajectories and time-varying mortality risk

**DOI:** 10.3389/fcimb.2026.1871653

**Published:** 2026-07-22

**Authors:** Baoyan Wang, Peijuan Xu, Jialin Qi, Qian Ni, Dan Han, Tong Qiao

**Affiliations:** 1 Nanjing Drum Tower Hospital, Affiliated Hospital of Medical School, Nanjing University, Nanjing, Jiangsu, China; 2 Nanjing Tongren Hospital, School of Medicine, Southeast University, Nanjing, Jiangsu, China; 3 Nanjing Drum Tower Hospital, Nanjing Drum Tower Hospital Clinical College, Nanjing University of Chinese Medicine, Nanjing, Jiangsu, China

**Keywords:** biomarker, body temperature, dynamic prediction, precision medicine, sepsis

## Abstract

**Background:**

Body temperature is a readily available physiological signal that may reflect dynamic host responses in sepsis, yet most studies rely on single or early measurements and do not capture the dynamic nature of thermoregulation during critical illness. Whether the association between body temperature and mortality varies over the course of sepsis remains unclear.

**Methods:**

In this multicenter retrospective cohort study, patients with sepsis were identified from the MIMIC-IV database as the derivation cohort and externally validated using the eICU Collaborative Research Database and a single-center intensive care unit (ICU) cohort from Nanjing Drum Tower Hospital. Body temperature was summarized into 6-hour intervals during the ICU stay. The primary outcome was 28-day in-hospital mortality. Joint latent class mixed models were used to identify temperature trajectory classes and generate landmark-based dynamic survival predictions. Piece-wise exponential additive mixed models were applied to estimate the nonlinear and time-varying association between infection-related temperature responses and in-hospital mortality.

**Results:**

A total of 8681 patients were included in the derivation cohort and 13717 in the validation cohort. Five distinct temperature trajectory classes were identified, demonstrating heterogeneous thermoregulatory patterns during the ICU stay. Mortality risk differed significantly across trajectory classes. The low-increase-moderate hyperthermia trajectory was associated with the highest mortality risk in both early and later periods of hospitalization. Dynamic prediction models incorporating longitudinal temperature trajectories showed better overall discrimination than models based on single temperature measurements. Time-varying analysis revealed a nonlinear U-shaped relationship between temperature and mortality, with the lowest risk near the upper range of normothermia. Both hypothermia and hyperthermia were associated with increased mortality, and the prognostic impact of infection-related temperature responses varied over the ICU course, peaking at approximately 4 days after ICU admission.

**Conclusion:**

Body temperature may serve as a dynamic host-response marker in sepsis, with prognostic significance determined by both temporal trajectory patterns and disease stage. Longitudinal temperature monitoring could provide a clinically accessible strategy for dynamic risk stratification and future temperature-guided precision management in sepsis.

## 1 Introduction

Sepsis remains a major cause of mortality worldwide and is characterized by marked heterogeneity in host response, disease progression, and treatment responsiveness. One challenge in risk stratification and individualized management is the lack of readily available markers that capture dynamic clinical states during critical illness (Scherger and Kalil, 2024; Antcliffe et al., 2025). Although molecular biomarkers have been extensively investigated, many remain costly, delayed, or impractical for real-time bedside use. Body temperature is one of the most frequently measured physiological variables in critically ill patients and reflects the complex interplay between host immune response, metabolic regulation, and disease severity (Drewry and Mohr, 2022). As such, temperature may represent a clinically accessible host-response biomarker (Zhao and Zhang, 2024; Inghammar and Sunden-Cullberg, 2020). However, most previous studies have relied on static or early measurements, potentially overlooking clinically important temporal patterns.

To overcome the limitations of static temperature measures, Bhavani and colleagues applied longitudinal temperature trajectory modeling to identify reproducible sepsis subphenotypes with distinct mortality risks (Bhavani et al., 2019, Bhavani et al., 2020, Bhavani et al., 2024). These trajectories were subsequently shown to be associated with divergent immune and coagulation profiles, supporting their biological relevance. Together, these findings highlight temperature dynamics as an important marker of heterogeneity in host response during sepsis. While temperature trajectory modeling has substantially advanced phenotyping efforts in sepsis (Kijpaisalratana et al., 2025; Bhavani et al., 2022), important gaps remain. First, trajectory-based approaches primarily classify patients according to early patterns but do not directly quantify how the prognostic impact of temperature evolves over time. Second, they provide limited insight into whether the mortality risk associated with abnormal temperature is constant throughout the intensive care unit (ICU) course or amplified during specific high-risk periods. As a result, the temporal interaction between thermoregulatory dynamics and evolving baseline mortality risk remains incompletely understood.

In the present study, we combined joint latent class mixed models to characterize temperature trajectories during the ICU stay with piece-wise exponential additive mixed models to evaluate the time-varying association between body temperature and in-hospital mortality in adult ICU patients with sepsis. This integrative approach allowed us to evaluate temperature trajectories within a dynamic risk framework and to examine whether deviations from normothermia were associated with periods of heightened mortality risk during the ICU course.

## 2 Materials and methods

### 2.1 Study design and populations

This study was a multicenter retrospective cohort analysis based on data from the Medical Information Mart for Intensive Care IV (MIMIC-IV) v3.1 database available at PhysioNet (certification number: 62316327), the eICU Collaborative Research Database (eICU-CRD) v2.0 and a single-center ICU cohort from Nanjing Drum Tower Hospital. Use of the MIMIC-IV and eICU-CRD databases was approved by the institutional review boards of the Massachusetts Institute of Technology and participating institutions, with a waiver of informed consent due to de-identified data. The Nanjing Drum Tower Hospital cohort was approved by the hospital ethics committee, and the requirement for informed consent was waived owing to the retrospective study design.

Patients with sepsis were identified separately in each data source according to dataavailability and clinical documentation practices. Patients were excluded if they met any of the following criteria: (1) age <18 years at ICU admission; (2) repeated ICU admissions during the same hospitalization (only the first ICU stay was retained); (3) fewer than five valid temperature measurements during the ICU stay, to ensure sufficient longitudinal information for reliable trajectory estimation. A detailed flowchart of patient selection and cohort construction is provided in **Supplementary Figure 1**. In the MIMIC-IV and eICU-CRD, sepsis was identified using International Classification of Diseases, Ninth and Tenth Revision (ICD-9 and ICD-10) diagnosis codes, consistent with previously published large database studies. These code-based algorithms have been widely used and validated for epidemiological research in administrative critical care databases. In contrast, in the Nanjing Drum Tower Hospital cohort, detailed clinical and laboratory data enabled the direct application of contemporary international diagnostic criteria. Sepsis was therefore defined according to the Sepsis-3 definition as suspected or confirmed infection accompanied by acute organ dysfunction, operationalized as an increase in Sequential Organ Failure Assessment (SOFA) score of ≥2 points (Singer et al., 2016). Diagnoses were adjudicated based on comprehensive review of electronic medical records, including clinical documentation, microbiological findings, and laboratory data.

### 2.2 Temperature measurement and preprocessing

Because the exact onset time of sepsis could not be reliably determined across databases, ICU admission was used as the common temporal anchor for all longitudinal analyses. Body temperature monitoring began at the time of ICU admission in all cohorts. Temperature measurements were extracted from routinely recorded bedside vital signs. To harmonize temporal resolution across databases, the ICU stay was divided into consecutive 6-hour intervals, and the maximum temperature value within each interval was retained to represent peak thermoregulatory response during that period. Using the maximum temperature within each interval allowed us to capture clinically relevant temperature elevations that may reflect the host inflammatory response. Temperature values were standardized to degrees Celsius.

### 2.3 Variable extraction

Baseline demographic characteristics, comorbidities, disease severity scores, laboratory tests, and physiological parameters were extracted from each database using standardized definitions. Demographic variables included age, sex, and race/ethnicity recorded at ICU admission. Pre-existing comorbidities were identified using International Classification of Diseases codes (ICD-9 or ICD-10) and included congestive heart failure (CHF), diabetes mellitus, hypertension, chronic pulmonary disease, malignancy, and chronic kidney disease (CKD). Overall comorbidity burden was summarized using the Charlson Comorbidity Index (CCI). Severity of illness at ICU admission was assessed using the Acute Physiology Score III (APS III), calculated according to established definitions using the worst physiological and laboratory values recorded within the first 24 hours after ICU admission. Laboratory variables included white blood cell count (WBC), hemoglobin concentration (Hb), lactate and platelet (PLT) extracted from routine blood tests performed within the first 24 hours after ICU admission. When multiple measurements were available, the first recorded value was used to represent baseline laboratory status. Physiological variables included respiratory rate, heart rate, peripheral oxygen saturation (SpO_2_), and mean arterial pressure (MAP). For baseline adjustment, the first recorded values within the first 24 hours after ICU admission were used. Organ support therapies included the use of mechanical ventilation and continuous renal replacement therapy (CRRT) during the ICU stay. All covariates were prespecified based on clinical relevance and prior literature and were included in multivariable models to adjust for potential confounding.

### 2.4 Outcomes

The primary outcome was 28-day in-hospital mortality, defined as death occurring during hospitalization within 28 days after ICU admission. Patients who were discharged alive before day 28 were treated as experiencing a competing event, as they were no longer at risk of in-hospital death. Patients remaining hospitalized beyond 28 days were administratively censored at day 28. This outcome was selected to ensure consistency and comparability across data sources, as post-discharge mortality information is not reliably available in the eICU-CRD database.

Hospital discharge vital status was obtained from discharge disposition variables in the MIMIC-IV and eICU-CRD cohorts and from electronic medical records in the Nanjing Drum Tower Hospital cohort. By focusing on in-hospital mortality, we ensured consistent outcome ascertainment across all cohorts while minimizing potential misclassification due to incomplete post-discharge follow-up.

### 2.5 Statistical analysis

Model development was performed in the MIMIC-IV cohort, and external validation was conducted using the combined eICU-CRD and Nanjing Drum Tower Hospital cohorts.

#### 2.5.1 Trajectory modeling, dynamic prediction, and model evaluation

In the derivation cohort (MIMIC-IV), longitudinal body temperature data were modeled using joint latent class mixed models (JLCMMs) to identify distinct temperature trajectory subphenotypes. The longitudinal submodel was specified using natural cubic splines of time since ICU admission, with both intercept and time effects allowed to vary across latent classes. The survival submodel included pre-specified baseline covariates, and a Weibull baseline hazard function was assumed. To ensure model stability and avoid convergence to local optima, an initial single-class model (ng = 1) was first fitted to obtain starting values. Subsequently, models with increasing numbers of latent classes were estimated using a grid search procedure with multiple random initializations.

Temperature trajectories were modeled using class-specific mixed-effects models, with time since ICU admission treated as a continuous variable. All available temperature measurements during the ICU stay were used to capture the full longitudinal evolution of thermoregulatory responses throughout critical illness. The optimal number of latent classes was determined based on Bayesian information criterion (BIC), posterior class-membership probabilities, entropy, and clinical interpretability. Each class was required to contain at least 5% of the study population, and patients were assigned to the class with the highest posterior probability.

Trajectory patterns were visualized to illustrate the temporal evolution of body temperature across classes. Kaplan-Meier curves were constructed within a landmark framework to compare survival across trajectory groups. Survival differences were assessed using the log-rank test. Time-dependent hazard ratios (HRs) were further estimated using a piecewise Cox proportional hazards model, with the optimal cut point determined by the highest log-likelihood.

#### 2.5.2 Dynamic prediction and model discrimination

The fitted JLCMMs were used to generate dynamic predictions of survival probability from a given landmark time to subsequent time points during follow-up. Each day from ICU admission through day 14 was defined as a landmark time point. At each landmark time, predicted survival probabilities from the landmark time to day 28 were derived for patients who remained alive at the landmark time. Patients who experienced the outcome prior to a given landmark time were excluded from the analysis at that landmark, consistent with standard landmark analysis methodology.

Model discrimination was evaluated using time-dependent area under the receiver operating characteristic curve (AUC) within the landmark framework. At each landmark time, survival time was redefined from the landmark to day 28 or censoring. For the static model, only the temperature value measured at the landmark time was used for prediction, whereas for the dynamic model, survival probabilities were obtained from the joint latent class model incorporating longitudinal temperature trajectories. Confidence intervals were estimated using bootstrap resampling with 2000 iterations, and model comparisons were performed using permutation testing with 2000 repetitions.

#### 2.5.3 Time-varying survival analysis

To further characterize the continuous and time-dependent association between body temperature and mortality beyond discrete trajectory groups, piece-wise exponential additive mixed models (PAMMs) were applied in the MIMIC-IV cohort. Follow-up time was discretized into short intervals, and body temperature was treated as a time-varying exposure. All available temperature measurements during the ICU stay were incorporated into the time-varying exposure structure. Penalized splines were used to flexibly model the nonlinear relationship between temperature and mortality risk as well as its interaction with time since ICU admission. Baseline covariates were included as time-fixed confounders.

To further evaluate the impact of temperature abnormalities, four complementary exposure metrics were analyzed: continuous temperature, threshold-defined abnormalities, proportion of exposure time, and cumulative exposure burden. Hypothermia was defined as temperature ≤36 °C and hyperthermia as temperature ≥39 °C. The proportion of time spent in hypothermia or hyperthermia was modeled as the hazard ratio per 5% increase in exposure time. Cumulative exposure burden was calculated as the total accumulated exposure below 36 °C or at or above 39 °C. Adjusted hazard ratios and 95% confidence intervals were estimated using multivariable Cox proportional hazards models.

#### 2.5.4 External validation

External validation was performed using the combined eICU-CRD and Nanjing Drum Tower Hospital cohorts. Temperature data were processed using the same preprocessing strategy as in the derivation cohort. Posterior class-membership probabilities were estimated using model parameters derived from the MIMIC-IV cohort, and trajectory distributions and associated mortality risks were compared across cohorts. Time-varying temperature-mortality associations were also re-estimated using the same modeling framework to assess reproducibility.

#### 2.5.5 Sensitivity analyses

In the primary analysis, cause-specific hazard models were applied, with discharge alive before day 28 treated as censoring. To account for competing events, Fine-Gray subdistribution hazard models were used in sensitivity analyses, treating discharge as a competing risk. Consistency between the two approaches was used to assess robustness. Furthermore, we repeated the trajectory and time-varying analyses using 28-day mortality as the outcome in the MIMIC-IV cohort, where complete follow-up was available. Temperature trajectories were re-identified using JLCMMs, and time-varying associations were evaluated using PAMMs, following the same analytical framework as in the primary analysis.

Continuous variables were summarized as mean (standard deviation) or median (interquartile range), as appropriate, and categorical variables as counts and percentages. Missing values in covariates were handled using multiple imputation by chained equations (MICE). All models were prespecified based on clinical relevance and prior literature. All analyses were performed using RStudio software (version 2026.01.1 + 403).

## 3 Results

### 3.1 Baseline characteristics of study populations

The derivation cohort comprised 8681 patients with sepsis from the MIMIC-IV database, of whom 1469 (16.92%) died before hospital discharge. The validation cohort included 13029 patients from the eICU-CRD and 688 patients from Nanjing Drum Tower Hospital who met the eligibility criteria; overall, 2263 patients (16.50%) died during hospitalization. Baseline characteristics of patients in the derivation and validation cohorts were summarized in **Table 1**. Patient characteristics were broadly comparable between the derivation and validation cohorts, although several differences were observed. Patients in the derivation cohort had a higher comorbidity burden, as reflected by a higher CCI. Laboratory values and vital signs were generally similar across cohorts. The use of mechanical ventilation was more frequent in the derivation cohort, while the proportion of patients receiving CRRT was comparable between groups. Compared with included patients, excluded patients had a higher mortality rate and shorter ICU length of stay (**Supplementary Tables 1** and **2**).

**Table 1:** Baseline characteristics of the derivation and validation cohorts.

Items	Derivation cohort (n=8681)	Validation cohort (n=13717)
Age (years), median [IQR]	68.98 [57.74, 79.62]	67.00 [55.00, 78.00]
Gender, n (%)		
Male	4836 (55.71%)	7217 (52.61%)
Female	3845 (44.29%)	6500 (47.39%)
Race, n (%)		
White	5767 (66.43%)	10211 (74.44%)
Black/African American	915 (10.54%)	1211 (8.83%)
Asian/Hispanic/Other	915 (10.54%)	876 (6.39%)
Unknown	1084 (12.49%)	1419 (10.34%)
Comorbidity, n (%)		
CHF	2951 (33.99%)	1607 (11.72%)
Diabetes	3065 (35.31%)	2252 (16.42%)
Chronic pulmonary disease	1055 (12.15%)	1609 (11.73%)
Hypertension	3753 (43.23%)	1892 (13.79%)
Cancer	2709 (31.21%)	709 (5.17%)
CKD	2485 (28.63%)	1705 (12.43%)
CCI (median [IQR])	6.00 [4.00, 8.00]	4.00 [3.00, 6.00]
Illness severity (median [IQR])		
APS III	57.00 [44.00, 74.00]	54.00 [41.00, 72.00]
Laboratory Index (median [IQR])		
WBC	13.20 [8.40, 19.40]	13.10 [8.70, 18.80]
Lactate	2.00 [1.30, 3.10]	1.70 [1.10, 2.80]
PLT	185.00 [121.00, 266.00]	180.00 [121.00, 251.00]
Hb	10.20 [8.70, 11.70]	10.40 [8.90, 12.10]
Vital signs (median [IQR])		
Respiratory rate	21.00 [17.00, 25.00]	22.00 [18.00, 27.00]
MAP	76.00 [66.00, 88.00]	74.00 [63.00, 89.00]
Heart rate	96.00 [82.00, 112.00]	98.00 [83.00, 113.00]
SpO_2_	97.00 [94.00, 99.00]	97.00 [94.00, 99.00]
Mechanical Ventilation	4141 (47.70%)	4467 (32.57%)
CRRT	353 (4.07%)	464 (3.38%)
28-day in-hospital mortality, n (%)	1469 (16.92%)	2263 (16.50%)
Length of ICU stay (days), median [IQR]	4.58 [2.81, 9.25]	3.74 [2.17, 7.57]

### 3.2 Identification of subpopulations using the JLCMMs

The number of latent classes was selected based on model fit and classification quality. Model fit improved with increasing numbers of classes, as indicated by higher log-likelihood values and decreasing BIC, with the lowest BIC observed for the five-class model (**Supplementary Table 3**). Although entropy peaked in the four-class model and declined slightly in the five-class model, classification quality remained acceptable in the five-class model, with adequate class sizes (all >5%) and high mean posterior probabilities across classes (>0.70, **Supplementary Table 4**), supporting reliable class assignment. Moreover, the five-class solution provided clearer separation in mortality risk. Therefore, the five-class model was selected as the primary trajectory solution. Similarly, **Supplementary Tables 5 and 6** presented the model fit metrics and mean posterior probabilities for the validation cohort.

**Figure 1:**
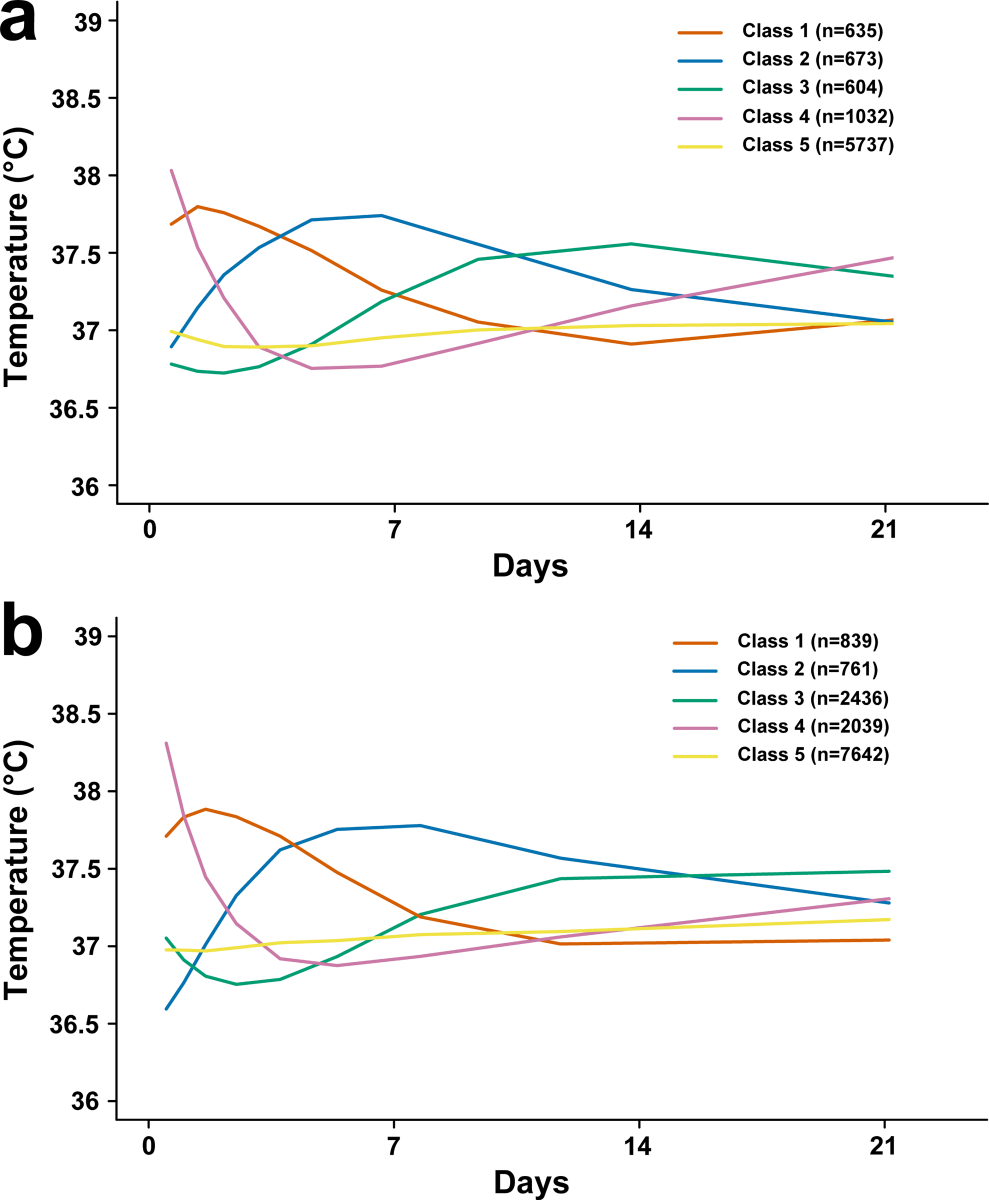
Temperature trajectory classes identified by joint latent class mixed models in the derivation and validation cohorts. **(a)** Model-estimated body temperature trajectories in the MIMIC-IV derivation cohort. **(b)** Corresponding temperature trajectory patterns in the external validation cohort. The x-axis represents time after ICU admission, and the y-axis represents body temperature in degrees Celsius.

Using joint latent class mixed models, five distinct temperature trajectories were identified during the ICU stay in the derivation cohort (**Figure 1a**), reflecting heterogeneous thermoregulatory patterns among patients. Class 1 (high-decrease-low plateau, n = 635) showed initially elevated temperatures with a progressive decrease during the early ICU course, stabilizing at lower levels thereafter. Class 2 (early peak-gradual decline, n = 673) displayed an early rise in temperature, peaking within the first week, followed by a gradual downward trend. Class 3 (low-increase-moderate hyperthermia, n = 604) demonstrated mildly reduced baseline temperatures followed by a gradual increase before slowly declining. Class 4 (high-decrease-rebound, n = 1032) was characterized by marked hyperthermia at ICU admission, followed by a rapid early decline and a gradual rebound over time. Class 5 (stable normothermia, n = 5737), the largest group, exhibited relatively stable normothermic temperatures with minimal fluctuation throughout follow-up. Overall, separation between trajectory classes became increasingly evident with longer ICU duration, indicating that longitudinal temperature patterns provided additional phenotypic information beyond early measurements alone. Comparable temperature trajectory patterns were observed in the validation cohort, demonstrating consistency of the identified trajectories across cohorts (**Figure 1b**).

**Figure 2:**
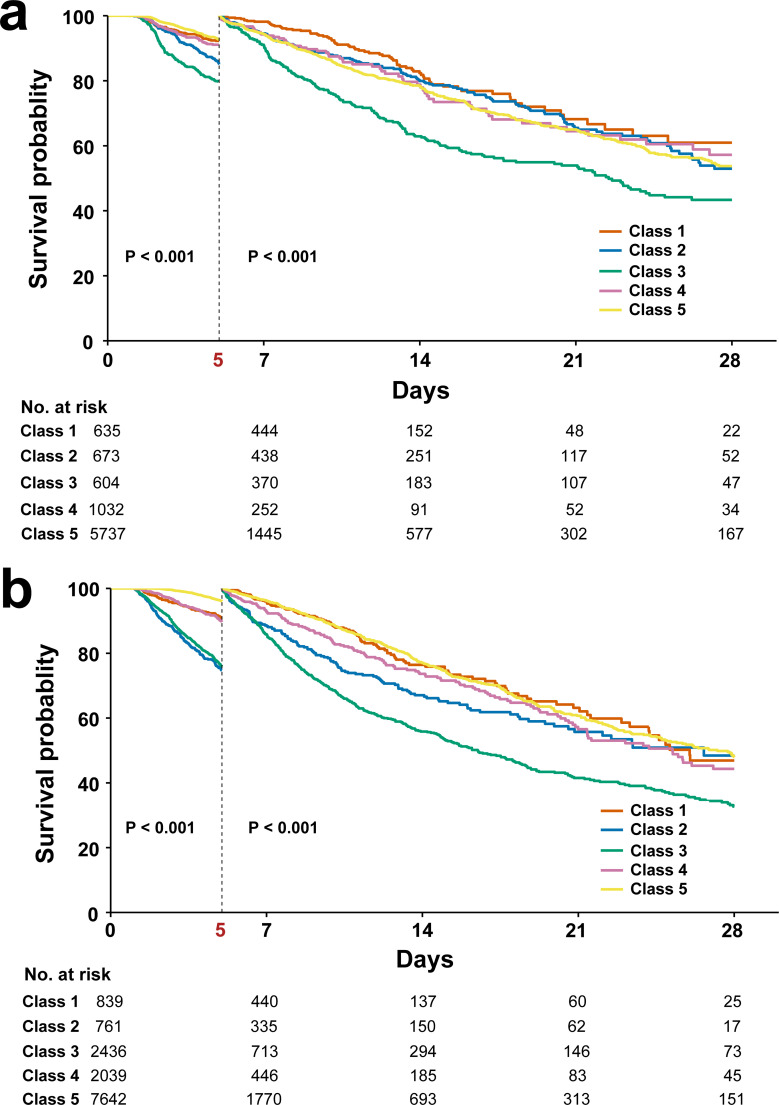
Kaplan-Meier curves for time to in-hospital mortality within 28 days after ICU admission, stratified by temperature trajectory class. **(a)** Survival curves in the MIMIC-IV derivation cohort. **(b)** Survival curves in the external validation cohort. Patients were grouped by temperature trajectory class, and survival differences were compared using the log-rank test; crossing curves supported subsequent piecewise Cox regression.

Landmark Kaplan-Meier survival curves demonstrated significant differences in in-hospital mortality among the five temperature trajectory classes in the derivation cohort (log-rank test, P < 0.001; **Figure 2a**). Crossing survival curves indicated potential violation of the proportional hazards. A piecewise Cox regression was utilized to account for time-varying coefficients and the model with the highest log-likelihood was selected, determining day 5 as the optimal cut point. The results from the piecewise Cox model indicated marked time-dependent differences in mortality risk across temperature trajectory classes (**Supplementary Table 7**). During the early period (0–5 days), Class 3 exhibited the highest mortality risk compared with Class 1 (HR = 2.85, 95%CI: 2.03-3.99), followed by Class 2 (HR = 1.94, 95%CI: 1.37-2.75). In the later period (5–28 days), Class 3 remained associated with the highest mortality risk (HR = 2.21, 95%CI: 1.69-2.90). Similar class-specific survival separation was observed in the validation cohort (**Figure 2b**; **Supplementary Table 8**).

### 3.3 Trajectory-specific dynamic prediction of in-hospital mortality

**Figure 3:**
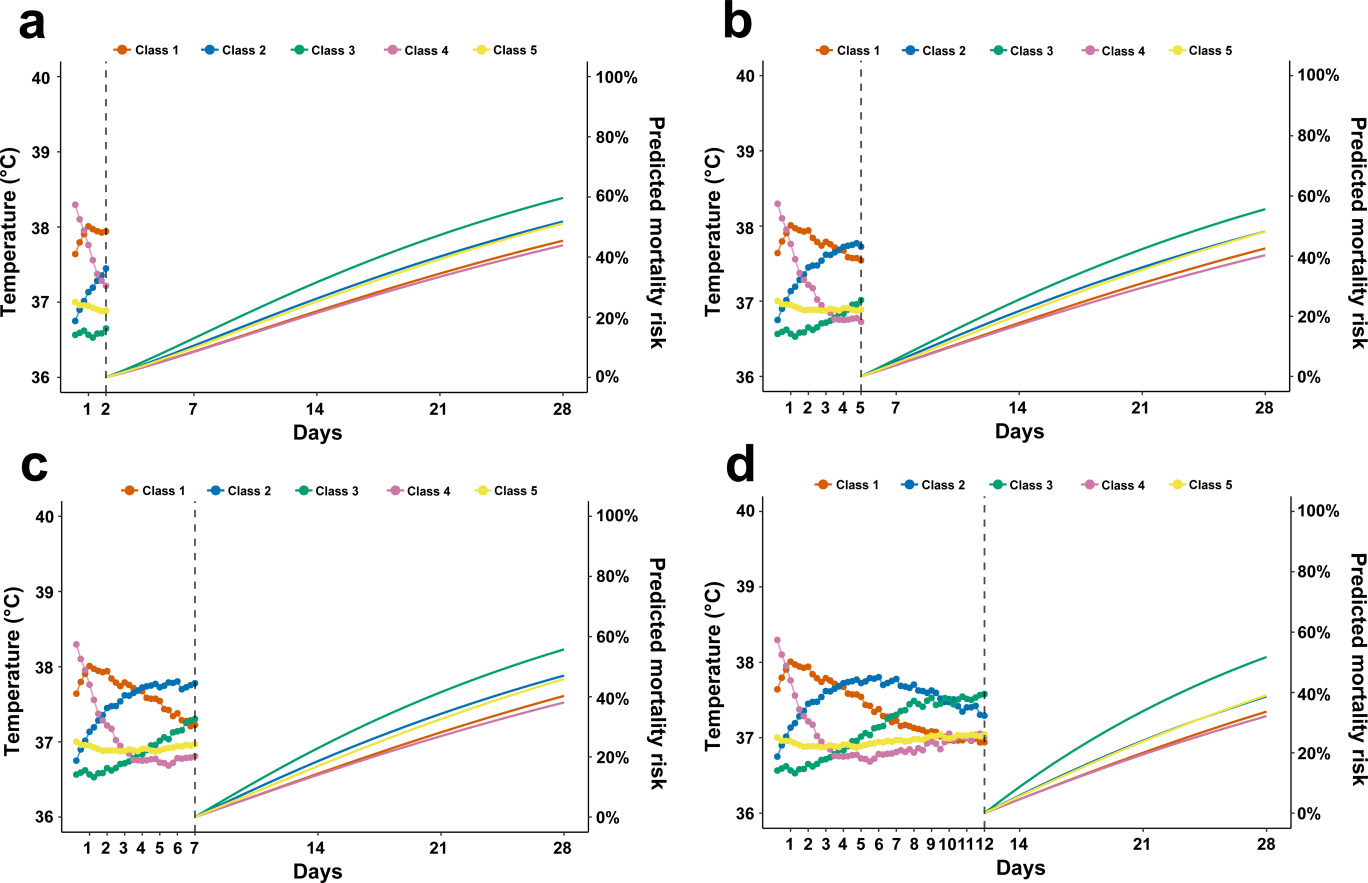
Dynamic body temperature trajectories and landmark-based prediction of 28-day in-hospital mortality across five trajectory classes in the derivation cohort. **(a)** Temperature measurements in the first 2 days and predicted mortality probability in the next 26 days; **(b)** Temperature measurements in the first 5 days and predicted mortality probability in the next 23 days; **(c)** Temperature measurements in the first 7 days and predicted mortality probability in the next 21 days; **(d)** Temperature measurements in the first 12 days and predicted mortality probability in the next 16 days.

**Figure 4:**
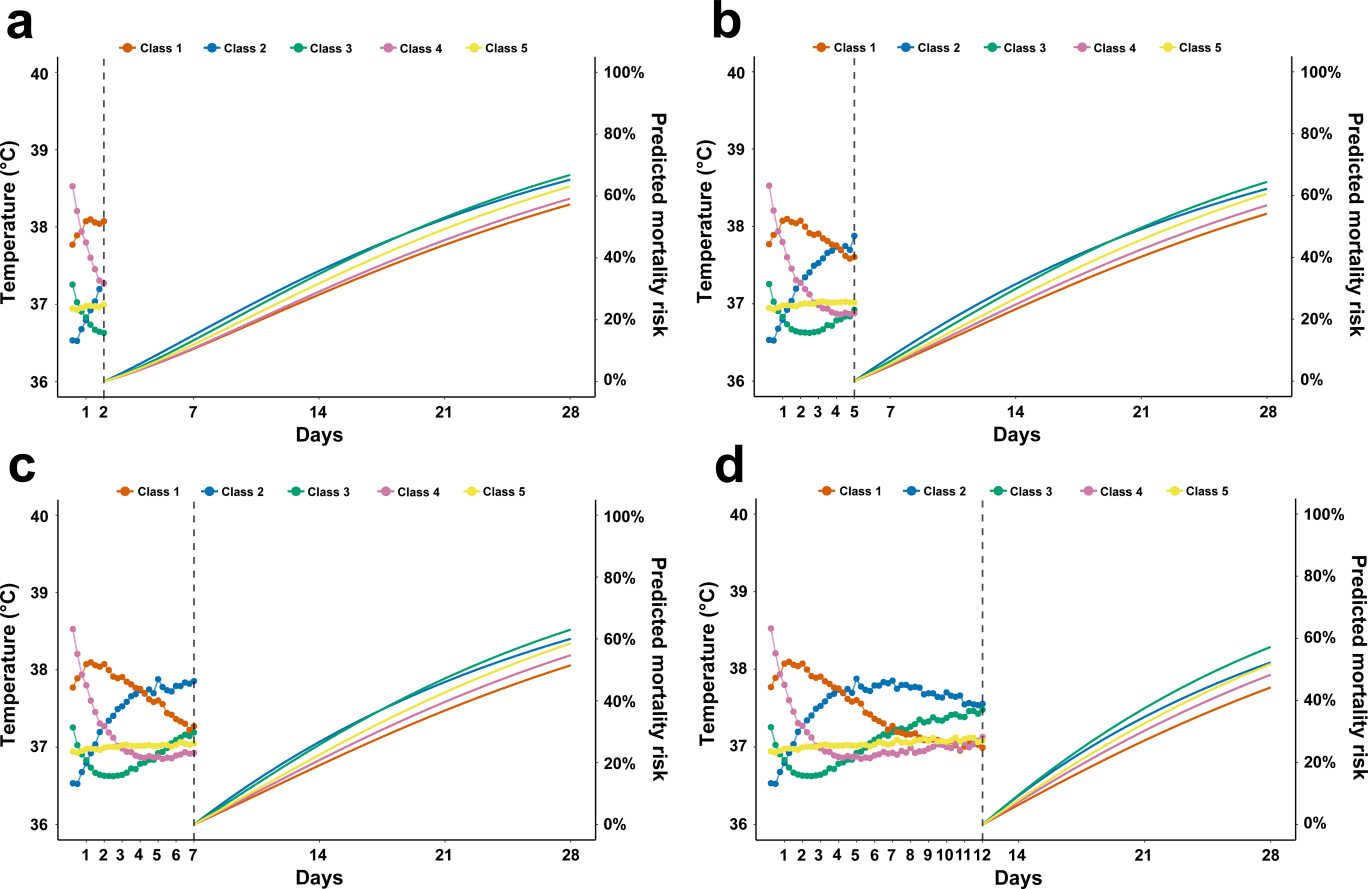
Dynamic body temperature trajectories and landmark-based prediction of 28-day in-hospital mortality across five trajectory classes in the validation cohort. **(a)** Temperature measurements in the first 2 days and predicted mortality probability in the next 26 days; **(b)** Temperature measurements in the first 5 days and predicted mortality probability in the next 23 days; **(c)** Temperature measurements in the first 7 days and predicted mortality probability in the next 21 days; **(d)** Temperature measurements in the first 12 days and predicted mortality probability in the next 16 days.

In the derivation cohort, clear separation in predicted mortality risk across trajectory classes was observed across landmark times (**Figure 3**). At the early landmark (day 2), Class 3 exhibited the highest predicted mortality, whereas Class 4 showed the lowest risk. The relative ranking of trajectory classes remained largely stable over time, while separation between high- and low-risk groups became more pronounced at later landmarks, particularly day 12, indicating that the prognostic value of temperature trajectories strengthened with accumulating longitudinal information. A similar pattern was observed in the external validation cohort (**Figure 4**). Notably, the stable normothermia trajectory (Class 5) remained at an intermediate risk level across most landmark times. These findings supported the robustness and generalizability of trajectory-based dynamic risk stratification.

Time-dependent AUCs were estimated within the landmark framework from day 1 to day 14 amongpatients alive at each landmark (**Supplementary Figure 2**). Both models showed moderate discrimination, with the dynamic model consistently outperforming the static model. Although statistical significance was not observed at every landmark, the dynamic model demonstrated superior discrimination across most early and intermediate time points and maintained an overall advantage throughout the 14-day period. These findings indicate that incorporating longitudinal temperature trajectories provides additional prognostic value beyond single time-point measurements.

### 3.4 Time-varying association between body temperature and in-hospital mortality

**Figure 5:**
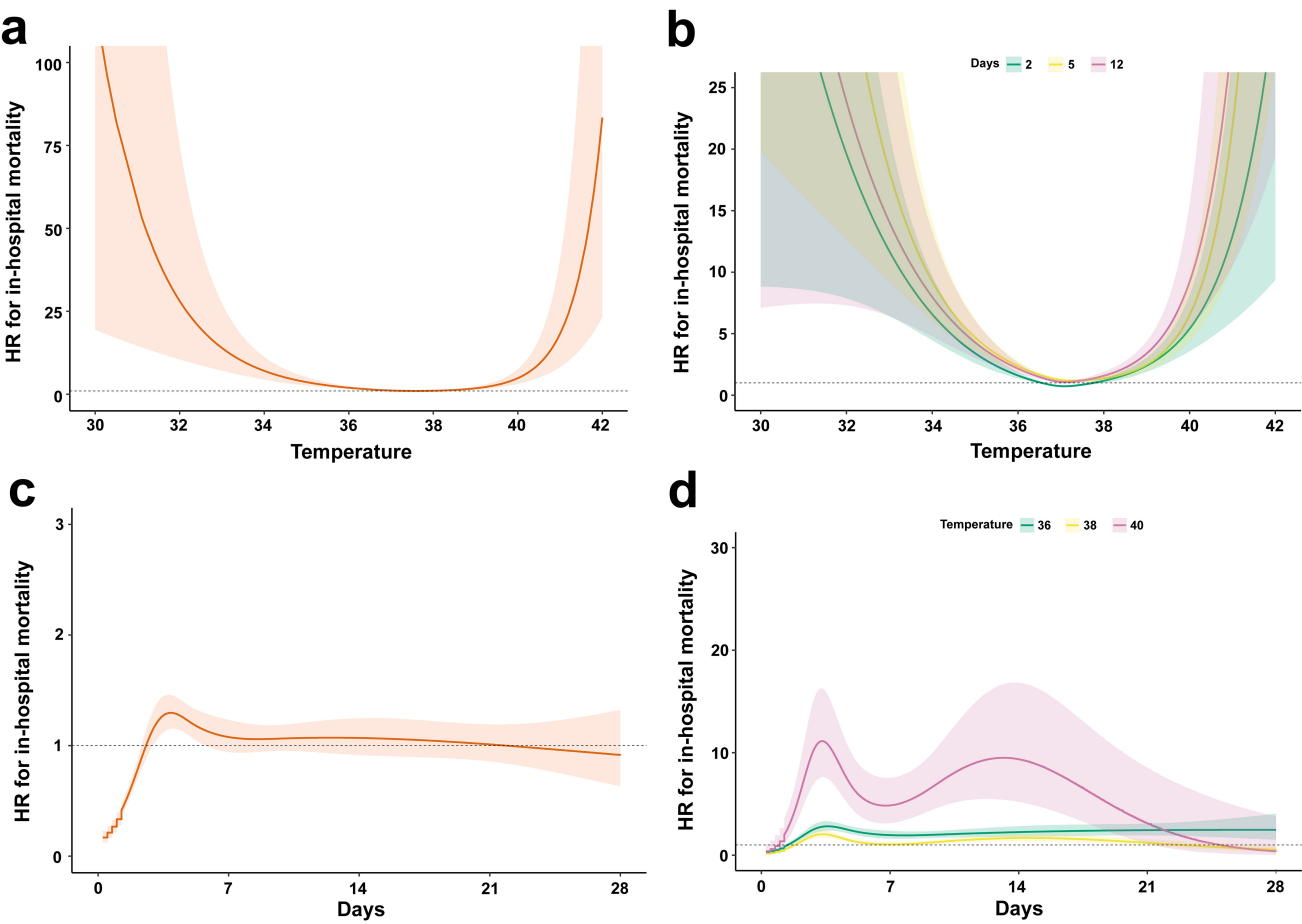
Time-varying association between body temperature and in-hospital mortality estimated using PAMMs in the derivation cohort. **(a)** Adjusted relationship between time-varying body temperature and in-hospital mortality; **(b)** Adjusted relationship between time-varying body temperature and in-hospital mortality stratified by landmark time points; **(c)** Time-varying effect of body temperature on in-hospital mortality; **(d)** Time-varying effect of body temperature on in-hospital mortality stratified by temperature levels.

**Figure 6:**
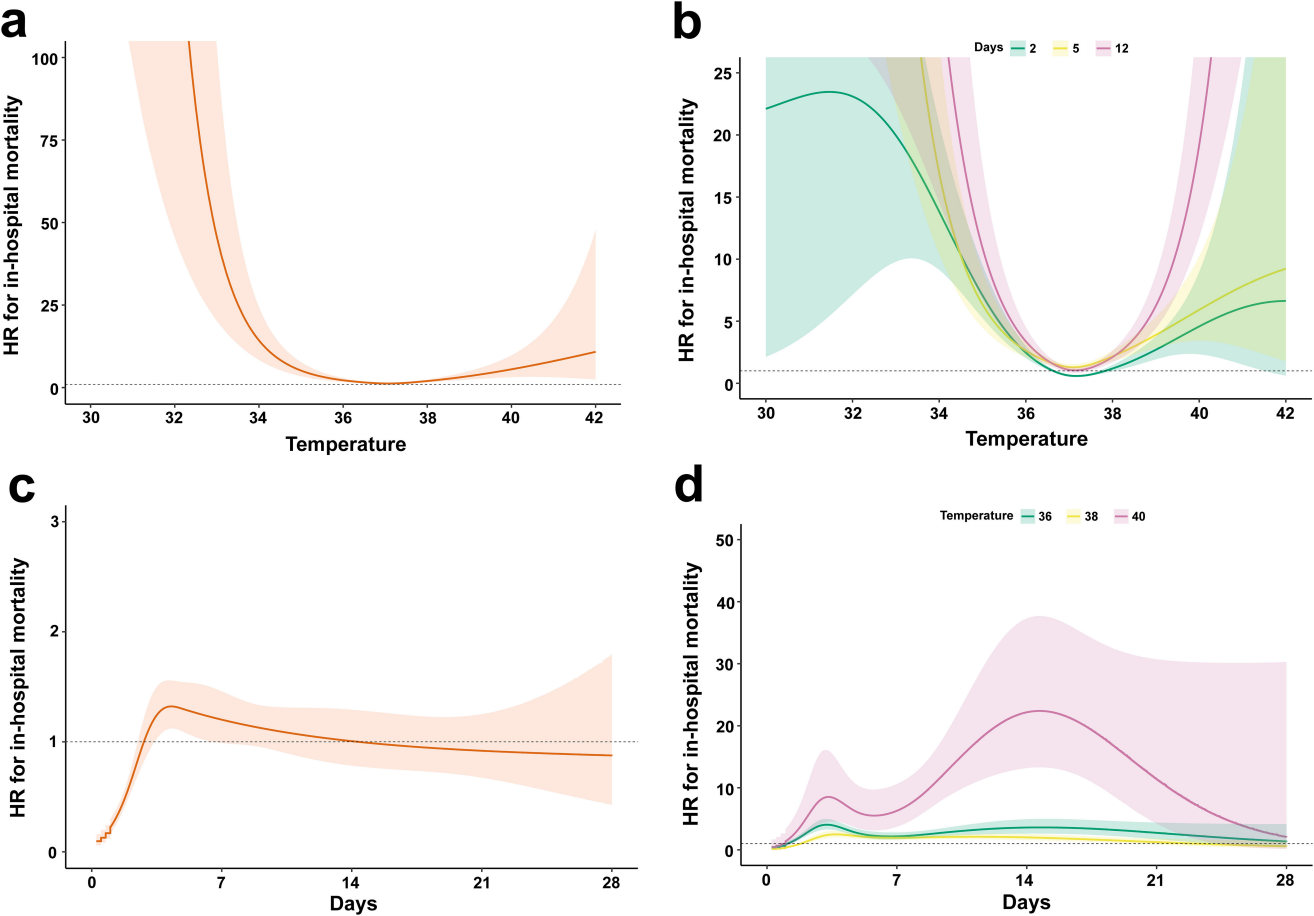
Time-varying association between body temperature and in-hospital mortality estimated using PAMMs in the validation cohort. **(a)** Adjusted relationship between time-varying body temperature and in-hospital mortality; **(b)** Adjusted relationship between time-varying body temperature and in-hospital mortality stratified by landmark time points; **(c)** Time-varying effect of body temperature on in-hospital mortality; **(d)** Time-varying effect of body temperature on in-hospital mortality stratified by temperature levels.

Using PAMMs, a non-linear U-shaped association was observed between time-varying temperature and mortality risk, with the lowest hazard observed at temperatures around 37.6 °C, near the upper range of normothermia. Both lower and higher temperatures were associated with increased risk (**Figure 5a**). When stratified by landmark time points (**Figure 5b**), the temperature-mortality association remained non-linear across all time windows. Temporal differences were most evident near the nadir of the curve, whereas the associations at lower and higher temperature ranges were more consistent across time. Mortality risk increased rapidly during the early phase after ICU admission, peaked at around day 4, and gradually attenuated thereafter (**Figure 5c**), indicating a time-dependent effect of temperature. When stratified by temperature levels, patients with hypothermia or hyperthermia exhibited greater temporal variation in mortality risk, whereas those with temperatures around the nadir of the U-shaped curve demonstrated the least fluctuation in risk over time (**Figure 5d**). Collectively, these findings demonstrated a dynamic and time-dependent relationship between body temperature and in-hospital mortality, supporting temperature as a clinically meaningful time-varying prognostic marker. Similar temporal patterns were observed in the external validation cohort, indicating good reproducibility of the temperature-mortality association across independent datasets (**Figure 6**).

Four complementary analyses consistently demonstrated significant associations between temperature abnormalities and mortality (**Supplementary Table 9**). When temperature was analyzed as a continuous variable, each 1 °C increase was associated with a modest reduction in mortality risk. Using prespecified, clinically defined thresholds, hypothermia (≤36 °C) and hyperthermia (≥39 °C) were defined in accordance with commonly used definitions in critical care literature (Laupland et al., 2008; O'Grady et al., 2008), and both were associated with increased mortality, consistent with the nonlinear temperature-mortality relationship observed in the primary analysis. Similarly, greater proportions of time spent in hypothermia or hyperthermia were associated with progressively higher mortality risk. Finally, cumulative exposure to temperature abnormalities showed a dose-response association with mortality, with both cumulative hypothermia and hyperthermia burdens significantly increasing the hazard of death. Similar associations were observed in the validation cohort (**Supplementary Table 10**).

### 3.5 Sensitivity analyses

To account for the competing event of alive hospital discharge, we performed Fine-Gray competing-risk analyses (**Supplementary Table 11**). In these analyses, Classes 2 and 3 remained significantly associated with increasedmortality, and Class 3 consistently exhibited the highest risk. For Classes 4 and 5, the estimatesdiffered from those of the cause-specific Cox models, likely reflecting differences in discharge patterns and the impact of competing events. In the sensitivity analysis using 28-day mortality as the outcome, similar temperature trajectory patterns were identified, with five distinct subphenotypes demonstrating consistent longitudinal trends compared with the primary analysis (**Supplementary Figure 4**). Dynamic prediction analyses within the landmark framework showed that differences inpredicted mortality risk across trajectory classes were already evident at early time points andbecame progressively more pronounced with increasing landmark time (**Supplementary Figure 5**). Notably, Class 3 consistently demonstrated the highest predicted mortality risk across alllandmark time points. In addition, PAMM analyses using 28-day mortality as the outcome yieldedresults consistent with the primary analysis (**Supplementary Figure 6**).

## 4 Discussion

In this multicenter study, we demonstrated that body temperature may act as a dynamic host-response biomarker in sepsis, with prognostic value determined by both longitudinal thermoregulatory patterns and disease stage. Five reproducible temperature trajectory classes were identified across cohorts. The low-increase-moderate hyperthermia trajectory consistently showed the highest mortality risk, whereas the stable normothermic trajectory was not consistently associated with the lowest risk. Incorporating trajectory information into a landmark-based framework improved dynamic risk discrimination compared with models based on single measurements. In parallel, time-varying analyses revealed a nonlinear U-shaped association between temperature and mortality, with the lowest risk near the upper range of normothermia and the strongest prognostic effect occurring during the early-to-middle phase after ICU admission. These findings suggested that temperature in sepsis could be interpreted as an evolving biological signal whose clinical meaning changes over time.

Trajectory-based and time-varying approaches provided complementary insights into thermoregulatory heterogeneity and dynamic risk evolution. JLCMM-derived trajectories captured between-patient heterogeneity by identifying distinct thermoregulatory phenotypes, whereas PAMMs quantified how the association between temperature and mortality changed during the course of illness. This distinction is clinically important because the same temperature value may carry different prognostic implications depending on when it occurs and how temperature has evolved previously. Conventional threshold-based approaches, which treat temperature as a static measurement, cannot capture this complexity. Our results suggested that risk assessment may benefit from considering both current temperature and preceding temperature trajectory.

Our findings should be interpreted in the context of previous studies on temperature abnormalities and outcomes in sepsis. Prior observational studies and meta-analyses have consistently reported that hypothermia was associated with increased mortality, whereas the prognostic meaning of fever has been more heterogeneous (Rumbus et al., 2017; Tan et al., 2024; Sundén-Cullberg et al., 2017). Moderate fever may reflect a preserved host response in selected patients, while excessive or persistent hyperthermia may indicate uncontrolled inflammation or metabolic stress. These apparently divergent findings may partly reflect differences in study populations, timing of temperature assessment, definitions of fever and hypothermia, and whether temperature was analyzed as a single measurement, a threshold-based exposure, or a longitudinal process. Our results helped refine this interpretation by showing that the prognostic meaning of temperature depends not only on its absolute level, but also on its trajectory and timing during the ICU course. In the PAMM analysis, the temperature-mortality association was nonlinear, with the lowest risk near the upper range of normothermia, whereas both hypothermia and hyperthermia were associated with increased risk. Importantly, this association was not constant over time, with the strongest prognostic impact observed during the early-to-middle phase after ICU admission. These findings suggested that fever, hypothermia, and normothermia should not be interpreted as fixed prognostic states; rather, their clinical implications may vary according to disease stage and longitudinal temperature evolution. Recent trajectory-based studies have shown that longitudinal temperature patterns identify reproducible sepsis subphenotypes associated with distinct clinical outcomes and immune or coagulation profiles (Zhang et al., 2025; Thomas-Rüddel et al., 2021). Our study was consistent with this concept but extended it by integrating trajectory phenotyping, landmark-based dynamic prediction, and time-varying hazard modeling. This framework showed that temperature dynamics provided prognostic information across multiple dimensions, including absolute temperature level, cumulative exposure, trajectory direction, and temporal context. It may also help explain why stable normothermia was not consistently associated with the lowest mortality risk, as normothermia may represent different underlying states, including true physiological stability, treated fever, impaired febrile response, or recovery from prior temperature abnormalities.

The observed temporal variability in the prognostic impact of temperature likely reflects evolving interactions among immune regulation, inflammation, and metabolic stress. Fever is a hallmark of host defense against infection, and moderate temperature elevation may inhibit pathogen replication, enhance immune activation, and promote effective host responses (Evans et al., 2015; Launey et al., 2011). Accordingly, early fever may indicate a relatively preserved adaptive immune response. In contrast, hypothermia may reflect impaired immune activation or exhaustion of host-defense mechanisms, including suppressed cytokine responses, reduced leukocyte function, and features of immune paralysis, which may compromise pathogen clearance and contribute to early mortality (Bhavani et al., 2019). Excessive or prolonged hyperthermia may instead indicate uncontrolled systemic inflammation and metabolic dysregulation; sustained high temperatures can increase cardiac workload, oxygen consumption, fluid loss, and organ injury risk (Doman et al., 2023). Thus, the nonlinear temperature-mortality relationship may arise from the balance between immune insufficiency at low temperatures and inflammatory-metabolic injury at high temperatures. The relative contribution of these mechanisms may shift over time, which could partly explain why the prognostic implications of temperature differ across disease stages.

Our findings also underscore the limitations of relying solely on single time-point measurements in critical illness. Sepsis is a dynamic syndrome characterized by changes in immune status, hemodynamics, metabolism, and organ function over time. A measurement obtained at one time point may not fully capture this evolving clinical state. Body temperature has practical advantages because it is routinely measured, noninvasive, inexpensive, and repeatedly available in most clinical settings. Rather than replacing established biomarkers or clinical assessment, longitudinal temperature patterns may provide complementary information on the evolving host response (Drewry et al., 2017; Young et al., 2019). This approach may be clinically useful because it relies on routinely collected bedside data and does not require additional assays or specialized equipment.

This study has several limitations. First, as a retrospective observational analysis, residual confounding and reverse causation cannot be excluded. In particular, important time-varying interventions such as antipyretic use, temperature management, and sedation were not fully captured, potentially introducing indication bias. Second, temperature measurements may be subject to heterogeneity in measurement sites, devices, and recording frequency across databases. Although time-window aggregation was applied, this approach may obscure transient extremes or misclassify short-term temperature abnormalities. Third, differences in sepsis definitions, clinical documentation, and data granularity across cohorts may affect comparability. Reliable classification of sepsis etiology could not be consistently achieved across all datasets, particularly in eICU-CRD. Nevertheless, external validation supports the robustness of the main findings. In addition, the exact onset time of sepsis could not be reliably determined across the included databases. Variability in the interval between true sepsis onset and ICU admission may have introduced heterogeneity in baseline inflammatory status and thermoregulatory responses. Fourth, patients with insufficient temperature measurements were excluded, which may have preferentially removed individuals with very short ICU stays, including those who died early after admission. As a result, excluded patients exhibited higher mortality and shorter lengths of stay, potentially introducing survivorship bias and limiting the generalizability of the findings to patients with early critical illness trajectories. This pattern suggests that patients with very early death or brief ICU stays were underrepresented in the trajectory analyses. However, this selection is inherent to trajectory-based analyses, which require repeated longitudinal measurements. Finally, threshold-based and cumulative exposure analyses were designed to enhance clinical interpretability rather than identify optimal cutoffs, and should be interpreted as supportive of the primary nonlinear associations.

## 5 Conclusion

In conclusion, the prognostic significance of body temperature in adult ICU patients with sepsis was not defined by a single value, but reflected its nonlinear level, temporal dynamics, and longitudinal trajectory. Longitudinal temperature monitoring may offer a scalable and clinically implementable tool for risk stratification in sepsis. Further prospective studies are needed to determine whether trajectory-informed temperature monitoring can improve clinical decision-making or patient outcomes.

## Data Availability

The data used in this study were derived from the MIMIC-IV database, the eICU Collaborative Research Database, and a single-center cohort from Nanjing Drum Tower Hospital. Access to MIMIC-IV and eICU-CRD requires an approved application. The single-center data are not publicly available because of institutional and privacy restrictions but may be available from the corresponding author upon reasonable request and with appropriate ethical approval.

## References

[B1] Antcliffe D. B. Burrell A. Boyle A. J. Gordon A. C. McAuley D. F. Silversides J. (2025). Sepsis subphenotypes, theragnostics and personalized sepsis care. Intensive Care Med. 51, 756–768. doi: 10.1007/s00134-025-07873-6 40163135 PMC12055953

[B2] Bhavani S. V. Carey K. A. Gilbert E. R. Afshar M. Verhoef P. A. Churpek M. M. (2019). Identifying novel sepsis subphenotypes using temperature trajectories. Am. J. Respir. Crit. Care Med. 200, 327–335. doi: 10.1164/rccm.201806-1197OC 30789749 PMC6680307

[B3] Bhavani S. V. Semler M. Qian E. T. Verhoef P. A. Robichaux C. Churpek M. M. . (2022). Development and validation of novel sepsis subphenotypes using trajectories of vital signs. Intensive Care Med. 48, 1582–1592. doi: 10.1007/s00134-022-06890-z 36152041 PMC9510534

[B4] Bhavani S. V. Spicer A. Sinha P. Malik A. Lopez-Espina C. Schmalz L. . (2024). Distinct immune profiles and clinical outcomes in sepsis subphenotypes based on temperature trajectories. Intensive Care Med. 50, 2094–2104. doi: 10.1007/s00134-024-07669-0 39382693 PMC12674214

[B5] Bhavani S. V. Wolfe K. S. Hrusch C. L. Greenberg J. A. Krishack P. A. Lin J. . (2020). Temperature trajectory subphenotypes correlate with immune responses in patients with sepsis. Crit. Care Med. 48, 1645–1653. doi: 10.1097/CCM.0000000000004610 32947475 PMC7837282

[B6] Doman M. Thy M. Dessajan J. Dlela M. Do Rego H. Cariou E. . (2023). Temperature control in sepsis. Front. Med. (Lausanne) 10, 1292468. doi: 10.3389/fmed.2023.1292468 38020082 PMC10644266

[B7] Drewry A. M. Ablordeppey E. A. Murray E. T. Stoll C. R. T. Izadi S. R. Dalton C. M. . (2017). Antipyretic therapy in critically ill septic patients: a systematic review and meta-analysis. Crit. Care Med. 45, 806–813. doi: 10.1097/CCM.0000000000002285 28221185 PMC5389594

[B8] Drewry A. Mohr N. M. (2022). Temperature management in the ICU. Crit. Care Med. 50, 1138–1147. doi: 10.1097/CCM.0000000000005556 35426840 PMC9232928

[B9] Evans S. S. Repasky E. A. Fisher D. T. (2015). Fever and the thermal regulation of immunity: the immune system feels the heat. Nat. Rev. Immunol. 15, 335–349. doi: 10.1038/nri3843 25976513 PMC4786079

[B10] Inghammar M. Sunden-Cullberg J. (2020). Prognostic significance of body temperature in the emergency department vs the ICU in patients with severe sepsis or septic shock: a nationwide cohort study. PloS One 15, e0243990. doi: 10.1371/journal.pone.0243990 33373376 PMC7771849

[B11] Kijpaisalratana N. Hassan A. Peng B. El Ariss A. B. Samadian K. Mitragotri S. . (2025). Exploring temperature trajectories in emergency department sepsis patients. Am. J. Emerg. Med. 95, 235–242. doi: 10.1016/j.ajem.2025.07.001 40617026

[B12] Launey Y. Nesseler N. Mallédant Y. Seguin P. (2011). Clinical review: fever in septic ICU patients--friend or foe? Crit. Care 15, 222. doi: 10.1186/cc10097 21672276 PMC3218963

[B13] Laupland K. B. Shahpori R. Kirkpatrick A. W. Ross T. Gregson D. B. Stelfox H. T. (2008). Occurrence and outcome of fever in critically ill adults. Crit. Care Med. 36, 1531–1535. doi: 10.1097/CCM.0b013e318170efd3 18434882

[B14] O'Grady N. P. Barie P. S. Bartlett J. G. Bleck T. Carroll K. Kalil A. C. . (2008). Guidelines for evaluation of new fever in critically ill adult patients: 2008 update from the American College of Critical Care Medicine and the Infectious Diseases Society of America. Crit. Care Med. 36, 1330–1349. doi: 10.1097/CCM.0b013e318169eda9 18379262

[B15] Rumbus Z. Matics R. Hegyi P. Zsiboras C. Szabo I. Illes A. . (2017). Fever is associated with reduced, hypothermia with increased mortality in septic patients: a meta-analysis of clinical trials. PloS One 12, e0170152. doi: 10.1371/journal.pone.0170152 28081244 PMC5230786

[B16] Scherger S. J. Kalil A. C. (2024). Sepsis phenotypes, subphenotypes, and endotypes: are they ready for bedside care? Curr. Opin. Crit. Care 30, 406–413. doi: 10.1097/MCC.0000000000001178 38847501

[B17] Singer M. Deutschman C. S. Seymour C. W. Shankar-Hari M. Annane D. Bauer M. . (2016). The third international consensus definitions for sepsis and septic shock (Sepsis-3). JAMA 315, 801–810. doi: 10.1001/jama.2016.0287 26903338 PMC4968574

[B18] Sundén-Cullberg J. Rylance R. Svefors J. Norrby-Teglund A. Björk J. Inghammar M. (2017). Fever in the emergency department predicts survival of patients with severe sepsis and septic shock admitted to the ICU. Crit. Care Med. 45, 591–599. doi: 10.1097/CCM.0000000000002249 28141683

[B19] Tan D. J. Chen J. Zhou Y. Ong J. S. Q. Sin R. J. X. Bui T. V. . (2024). Association of body temperature and mortality in critically ill patients: an observational study using two large databases. Eur. J. Med. Res. 29, 33. doi: 10.1186/s40001-023-01616-3 38184625 PMC10770998

[B20] Thomas-Rüddel D. O. Hoffmann P. Schwarzkopf D. Scheer C. Bach F. Komann M. . (2021). Fever and hypothermia represent two populations of sepsis patients and are associated with outside temperature. Crit. Care 25, 368. doi: 10.1186/s13054-021-03776-2 34674733 PMC8532310

[B21] Young P. J. Bellomo R. Bernard G. R. Niven D. J. Schortgen F. Saxena M. . (2019). Fever control in critically ill adults. An individual patient data meta-analysis of randomised controlled trials. Intensive Care Med. 45, 468–476. doi: 10.1007/s00134-019-05553-w 30741326

[B22] Zhang X. Zhang W. Zhang H. Liao X. L. (2025). Sepsis subphenotypes: bridging the gaps in sepsis treatment strategies. Front. Immunol. 16, 1546474. doi: 10.3389/fimmu.2025.1546474 40013154 PMC11862915

[B23] Zhao Y. N. Zhang B. (2024). Association between body temperature and all-cause mortality in patients with sepsis: analysis of the MIMIC-IV database. Eur. J. Med. Res. 29, 630. doi: 10.1186/s40001-024-02219-2 39726025 PMC11673708

