# Supplementary material for: Body temperature as a dynamic host-response biomarker in sepsis: trajectories and time-varying mortality risk
Source: Front Cell Infect Microbiol. 2026 Jul 22;16:1871653. doi: 10.3389/fcimb.2026.1871653 (PMC13437298; doi:10.3389/fcimb.2026.1871653)
Supplement: Supplementary file 1 [file DataSheet1.docx]

**Table S1** Included vs excluded baseline comparison in the derivation cohort

| **Items** | **Excluded**  **(n=2000)** | **Included**  **(n=8681)** | **SMD** |
| --- | --- | --- | --- |
| **Age (years), median [IQR]** | 70.82 [59.16, 82.14] | 68.98 [57.74, 79.62] | 0.121 |
| **Gender, n (%)** |  |  |  |
| Male | 1062 (53.10%) | 4836 (55.71%) | 0.052 |
| Female | 938 (46.90%) | 3845 (44.29%) |  |
| **Race****, n (%)** |  |  |  |
| White | 1300 (65.00%) | 5767 (66.43%) | 0.083 |
| Black/African American | 248 (12.40%) | 915 (10.54%) |  |
| Asian/Hispanic/Other | 235 (11.75%) | 915 (10.54%) |  |
| Unknown | 217 (10.85%) | 1084 (12.49%) |  |
| **Comorbidity, n (%)** |  |  |  |
| CHF | 587 (29.35%) | 2951 (33.99%) | 0.100 |
| Diabetes | 701 (35.05%) | 3065 (35.31%) | 0.005 |
| Chronic pulmonary disease | 208 (10.40%) | 1055 (12.15%) | 0.055 |
| Hypertension | 845 (42.25%) | 3753 (43.23%) | 0.02 |
| Cancer | 695 (34.75%) | 2709 (31.21%) | 0.075 |
| CKD | 522 (26.10%) | 2485 (28.63%) | 0.057 |
| **CCI (median [IQR])** | 6.00 [4.00, 8.00] | 6.00 [4.00, 8.00] | 0.012 |
| **Illness severity (median [IQR])** |  |  |  |
| APS Ⅲ | 52.00 [38.00, 79.00] | 57.00 [44.00, 74.00] | 0.020 |
| **Laboratory Index (median [IQR])** |  |  |  |
| WBC | 12.95 [8.00, 18.90] | 13.20 [8.40, 19.40] | 0.015 |
| Lactate | 2.40 [1.50, 4.60] | 2.00 [1.30, 3.10] | 0.022 |
| PLT | 180.00 [116.00, 262.00] | 185.00 [121.00, 266.00] | 0.055 |
| Hb | 10.20 [8.80, 11.70] | 10.20 [8.70, 11.70] | 0.015 |
| **Vital signs (median [IQR])** |  |  |  |
| Respiratory rate | 21.00 [17.00, 25.00] | 21.00 [17.00, 25.00] | 0.034 |
| MAP | 75.00 [66.00, 87.00] | 76.00 [66.00, 88.00] | 0.040 |
| Heart rate | 95.00 [81.00, 110.00] | 96.00 [82.00, 112.00] | 0.046 |
| SpO_2_ | 96.00 [94.00, 99.00] | 97.00 [94.00, 99.00] | 0.026 |
| **Mechanical Ventilation** | 518 (25.90%) | 4141 (47.70%) | 0.464 |
| **CRRT** | 82 (4.10%) | 353 (4.07%) | 0.002 |
| **In-hospital mortality, n (%)** | 685 (34.25%) | 1469 (16.92%) | 0.305 |
| **Length of ICU stay (days), median [IQR]** | 1.71 [1.30, 1.96] | 4.58 [2.81, 9.25] | 1.223 |

**Table S2** Included vs excluded baseline comparison in the validation cohort

| **Items** | **Excluded**  **(n=6609)** | **Included**  **(n=13717)** | **SMD** |
| --- | --- | --- | --- |
| **Age (years), median [IQR]** | 67.00 [55.00, 79.00] | 67.00 [55.00, 78.00] | 0.009 |
| **Gender, n (%)** |  |  |  |
| Male | 3383 (51.19%) | 7217 (52.61%) | 0.029 |
| Female | 3226 (48.81%) | 6500 (47.39%) |  |
| **Race, n (%)** |  |  |  |
| White | 4853 (73.43%) | 10211 (74.44%) | 0.111 |
| Black/African American | 635 (9.61%) | 1211 (8.83%) |  |
| Asian/Hispanic/Other | 291 (4.40%) | 876 (6.39%) |  |
| Unknown | 830 (12.56%) | 1419 (10.34%) |  |
| **Comorbidity, n (%)** |  |  |  |
| CHF | 497 (7.52%) | 1607 (11.72%) | 0.143 |
| Diabetes | 925 (14.00%) | 2252 (16.42%) | 0.067 |
| Chronic pulmonary disease | 664 (10.05%) | 1609 (11.73%) | 0.054 |
| Hypertension | 629 (9.52%) | 1892 (13.79%) | 0.134 |
| Cancer | 254 (3.84%) | 709 (5.17%) | 0.064 |
| CKD | 536 (8.11%) | 1705 (12.43%) | 0.143 |
| **CCI (median [IQR])** | 4.00 [3.00, 5.00] | 4.00 [3.00, 6.00] | 0.088 |
| **Illness severity (median [IQR])** |  |  |  |
| APS Ⅲ | 57.00 [43.00, 75.00] | 54.00 [41.00, 72.00] | 0.325 |
| **Laboratory Index (median [IQR])** |  |  |  |
| WBC | 12.30 [8.10, 17.70] | 13.10 [8.70, 18.80] | 0.035 |
| Lactate | 1.80 [1.10, 3.10] | 1.70 [1.10, 2.80] | 0.186 |
| PLT | 181.00 [126.00, 249.00] | 180.00 [121.00, 251.00] | 0.010 |
| Hb | 10.50 [9.10, 11.90] | 10.40 [8.90, 12.10] | 0.224 |
| **Vital signs (median [IQR])** |  |  |  |
| Respiratory rate | 22.00 [18.00, 27.00] | 22.00 [18.00, 27.00] | 0.015 |
| MAP | 75.00 [64.00, 88.00] | 74.00 [63.00, 89.00] | 0.006 |
| Heart rate | 96.00 [82.00, 111.00] | 98.00 [83.00, 113.00] | 0.078 |
| SpO_2_ | 97.00 [94.00, 99.00] | 97.00 [94.00, 99.00] | 0.007 |
| **Mechanical Ventilation** | 660 (9.99%) | 4467 (32.57%) | 0.574 |
| **CRRT** | 49 (0.74%) | 464 (3.38%) | 0.187 |
| **In-hospital mortality, n (%)** | 1264 (19.17%) | 2263 (16.50%) | 0.070 |
| **Length of ICU stay (days), median [IQR]** | 0.93 [0.64, 1.54] | 3.74 [2.17, 7.57] | 0.920 |

**Table S3** Model fit metrics for determining the optimal number of classes in the derivation cohort

| **No. of classes** | **Log likelihood** | **BIC** | **Entropy** | **Class 1** | **Class 2** | **Class 3** | **Class 4** | **Class 5** |
| --- | --- | --- | --- | --- | --- | --- | --- | --- |
| 1 | -217239 | 434649.6 | 1.000000 | 100 |  |  |  |  |
| 2 | -211850 | 423936.1 | 0.478839 | 42.95588 | 57.04412 |  |  |  |
| 3 | -208317 | 416934.0 | 0.611610 | 66.54763 | 17.68229 | 15.77007 |  |  |
| 4 | -206556 | 413475.4 | 0.612452 | 13.60442 | 14.88308 | 64.84276 | 6.669739 |  |
| 5 | -205274 | 410974.4 | 0.611267 | 7.314825 | 7.752563 | 6.957724 | 11.88803 | 66.08686 |

**Table S4** Mean posterior probabilities for candidate latent class solutions in the derivation cohort

| **No. of classes** | **Class 1** | **Class 2** | **Class 3** | **Class 4** | **Class 5** |
| --- | --- | --- | --- | --- | --- |
| 1 | 1.000000 |  |  |  |  |
| 2 | 0.831683 | 0.813463 |  |  |  |
| 3 | 0.809760 | 0.855414 | 0.851371 |  |  |
| 4 | 0.838239 | 0.808290 | 0.764788 | 0.837429 |  |
| 5 | 0.820264 | 0.836667 | 0.760835 | 0.823471 | 0.718832 |

**Table S5** Model fit metrics for determining the optimal number of classes in the validation cohort

| **No. of classes** | **Log likelihood** | **BIC** | **Entropy** | **Class 1** | **Class 2** | **Class 3** | **Class 4** | **Class 5** |
| --- | --- | --- | --- | --- | --- | --- | --- | --- |
| 1 | -268713 | 537587.2 | 1 | 100 |  |  |  |  |
| 2 | -260987 | 522203.3 | 0.6474112 | 73.5292 | 26.4708 |  |  |  |
| 3 | -257198 | 514691.7 | 0.6202363 | 66.80032 | 14.66064 | 18.53904 |  |  |
| 4 | -255014 | 510389.6 | 0.5694762 | 21.60093 | 5.504119 | 57.35219 | 15.54276 |  |
| 5 | -253409 | 507247.2 | 0.5756488 | 6.116498 | 5.54786 | 17.75899 | 14.86477 | 55.71189 |

**Table S6** Mean posterior probabilities for candidate latent class solutions in the validation cohort

| **No. of classes** | **Class 1** | **Class 2** | **Class 3** | **Class 4** | **Class 5** |
| --- | --- | --- | --- | --- | --- |
| 1 | 1.000000 |  |  |  |  |
| 2 | 0.9067756 | 0.8705667 |  |  |  |
| 3 | 0.8170913 | 0.8237419 | 0.8637889 |  |  |
| 4 | 0.7230193 | 0.8182802 | 0.6996288 | 0.8444835 |  |
| 5 | 0.7809521 | 0.8120727 | 0.6457238 | 0.8182128 | 0.6475220 |

**Table S7** Time-dependent HR for four classes in the derivation cohort

| **Groups** | **HR 95%CI** | |
| --- | --- | --- |
|  | **(0, 5]** | **(5, 28]** |
| **Class 2** | 1.94 (1.37-2.75) | 1.31 (0.98-1.73) |
| **Class 3** | 2.85 (2.03-3.99) | 2.21 (1.69-2.90) |
| **Class 4** | 1.21 (0.84-1.75) | 1.32 (0.95-1.84) |
| **Class 5** | 0.88 (0.64-1.20) | 1.43 (1.12-1.83) |

**Table S8** Time-dependent HR for four classes in the validation cohort

| **Groups** | **HR 95%CI** |  |
| --- | --- | --- |
|  | **(0, 5]** | **(5, 28]** |
| **Class 2** | 3.03 (2.28-4.02) | 1.45 (1.13-1.87) |
| **Class 3** | 2.84 (2.20-3.67) | 2.11 (1.71-2.61) |
| **Class 4** | 1.05 (0.78-1.42) | 1.24 (0.97-1.58) |
| **Class 5** | 0.33 (0.24-0.44) | 1.02 (0.82-1.25) |

**Table S9** Associations between multiple temperature exposure metrics and in-hospital mortality in the derivation cohort

| **Item** | **Exposure metric** | **Definition** | **HR (95% CI)** |
| --- | --- | --- | --- |
| **Continuous effect** | Temperature | Per 1°C increase | 0.92 (0.86-0.99) |
| **Threshold effect** | Hypothermia | Temperature ≤36℃ | 1.66 (1.51-1.84) |
|  | Hyperthermia | Temperature ≥39℃ | 1.38 (1.24-1.54) |
| **Exposure proportion** | Hypothermia proportion | Per 5% increase in time spent with temperature ≤36℃ | 1.05 (1.03-1.06) |
|  | Hyperthermia proportion | Per 5% increase in time spent with temperature ≥39℃ | 1.10 (1.07-1.13) |
| **Cumulative burden** | Hypothermia burden | Cumulative exposure ≤36℃ | 1.16 (1.09-1.23) |
|  | Hyperthermia burden | Cumulative exposure ≥39℃ | 1.42 (1.20-1.67) |

**Table S10** Associations between multiple temperature exposure metrics and in-hospital mortality in the validation cohort

| **Item** | **Exposure metric** | **Definition** | **HR (95% CI)** |
| --- | --- | --- | --- |
| **Continuous effect** | Temperature | Per 1°C increase | 0.85 (0.78-0.93) |
| **Threshold effect** | Hypothermia | Temperature ≤36℃ | 1.90 (1.67-2.17) |
|  | Hyperthermia | Temperature ≥39℃ | 1.57 (1.35-1.82) |
| **Exposure proportion** | Hypothermia proportion | Per 5% increase in time spent with temperature ≤36℃ | 1.09 (1.07-1.11) |
|  | Hyperthermia proportion | Per 5% increase in time spent with temperature ≥39℃ | 1.08 (1.05-1.13) |
| **Cumulative burden** | Hypothermia burden | Cumulative exposure ≤36℃ | 1.38 (1.29-1.48) |
|  | Hyperthermia burden | Cumulative exposure ≥39℃ | 1.27 (1.10-1.46) |

**Table S11** Association between temperature trajectory classes and in-hospital mortality:

comparison of cause-specific and competing-risk models

| **Item** | **Cause-specific hazard models** | **Fine-Gray competing-risk model** |
| --- | --- | --- |
|  | **HR (95%CI)** | **sHR (95%CI)** |
| **Class 2** | 1.44 (1.16-1.80) | 1.71 (1.37-2.14) |
| **Class 3** | 2.07 (1.67-2.55) | 2.22 (1.78-2.78) |
| **Class 4** | 1.17 (0.91-1.49) | 0.70 (0.54-0.90) |
| **Class 5** | 0.99 (0.82-1.20) | 0.57 (0.47-0.70) |

HR indicates cause-specific hazard ratio; sHR indicates subdistribution hazard ratio accounting for the competing event of alive hospital discharge.

**Figure S1** Flowchart of patient selection in the derivation and validation cohorts.

**Figure S2** Time-dependent AUC for two models at different landmark times in the derivation cohort **(a)** and validation cohort **(b)**. The static model used the temperature value measured at each landmark time, whereas the dynamic model incorporated longitudinal temperature trajectory information. AUCs were calculated from each landmark time to day 28 among patients who remained alive at the landmark. Error bars indicate bootstrap confidence intervals.

**Figure S3** Time-varying effect of body temperature on in-hospital mortality in the derivation cohort **(a)** and validation cohort **(b)**. The figure showed the estimated temporal variation in the temperature-mortality association during follow-up.

**Figure S4** Temperature trajectory classes identified by joint latent class modeling in the derivation cohort with 28-day mortality as outcome. The same joint latent class mixed modeling framework was applied using 28-day mortality as the outcome. Five trajectory classes with similar longitudinal patterns to the primary analysis were identified.

**Figure S5** Dynamic body temperature trajectories and landmark-based prediction of 28-day mortality across five trajectory classes in the derivation cohort. **(a)** Temperature measurements in the first 2 days and predicted mortality probability in the next 26 days; **(b)** Temperature measurements in the first 5 days and predicted mortality probability in the next 23 days; **(c)** Temperature measurements in the first 7 days and predicted mortality probability in the next 21 days; **(d)** Temperature measurements in the first 12 days and predicted mortality probability in the next 16 days.

**Figure S6** Time-varying association between body temperature and 28-day mortality estimated using PAMMs in the derivation cohort. **(a)** Adjusted relationship between time-varying body temperature and 28-day mortality; **(b)** Adjusted relationship between time-varying body temperature and 28-day mortality stratified by landmark time points; **(c)** Time-varying effect of body temperature on 28-day mortality; **(d)** Time-varying effect of body temperature on 28-day mortality stratified by temperature levels.
